# A deformylase inhibitor expands therapeutic options for Lyme disease

**DOI:** 10.21203/rs.3.rs-9335039/v1

**Published:** 2026-05-08

**Authors:** Kim Lewis, Pankaj Patil, Hsin-Wen Liang, Eleanor Astley, Raleb Taher, Mi-Hyun Lee, Daniel Norment, Akira Iinishi, Nikita Gupta, Katerina Sobolevskaia, Eric Chen, Akbar Nawab, Justice Adzre, Caitlin Moss, Bryson Hawkins, Jeffrey Bourgeois, Melissa Caimano, Catherine Brissette, Linden Hu

**Affiliations:** Northeastern University; Northeastern university; Northeastern university; Tufts university; Northeastern university; Northeastern university; Northeastern university; Antimicrobial Discovery Center, Department of Biology, Northeastern University; Northeastern university; Northeastern university; Tufts university; University of North Dakota; University of North Dakota; Yale university; University of New South Wales; Worcester Polytechnic Institute; University of Connecticut Health Center; University of North Dakota; Tufts Medicine

## Abstract

Lyme disease incidence continues to rise globally. This vector-borne infection remains a major public health burden. Broad-spectrum doxycycline and ceftriaxone disrupt the gut microbiome, drive resistance in commensals, and offer suboptimal efficacy against neuroborreliosis. Here we show that forazemin, previously known as BB-83698, is an orally bioavailable peptide deformylase inhibitor with potent and selective bactericidal activity against spirochaetes, including diverse *Borrelia* species. Targeting the deformylation of nascent peptides, forazemin halted protein synthesis, thereby killing the spirochaetes. In murine models of Lyme borreliosis and neuroborreliosis, short oral dosing regimens cleared infection, and forazemin was more effective than doxycycline in tick-bite prophylaxis. Forazemin preserved microbiome diversity and spared beneficial gut symbionts. These findings support forazemin as a candidate for the treatment and prevention of Lyme disease.

## Introduction

Lyme disease, also known as Lyme borreliosis, is the most commonly reported vector-borne infection in the Northern Hemisphere and remains a major public health challenge^1–4^. In the United States, recent estimates suggest that nearly 500,000 new cases occur each year, making it one of the most prevalent bacterial infections^5^. The economic impact of this burden is considerable, with direct healthcare costs approaching $1.2 billion annually and additional losses tied to reduced workforce productivity^6^. Several factors drive the rising incidence of Lyme disease, including expansion of the geographic range of ticks carrying *Borrelia*, increasing overlap of human residential areas with habitats of reservoir hosts, and longer seasonal activity associated with climate change^7,8^. These changes have contributed both to the rising number of cases and to the growing distribution of infections with *Borreliella burgdorferi* (formerly *Borrelia burgdorferi*)^7,8^.

Acute Lyme disease typically presents with an erythema migrans rash at the site of the tick bite, a hallmark clinical feature of early infection^4^. Without prompt treatment, *B. burgdorferi* rapidly disseminates to additional skin sites and can invade the heart, peripheral nerves, and central nervous system, leading to manifestations such as radiculopathy, cranial nerve palsies, and meningitis^4^. If left untreated, infection can progress to late-stage disease characterized by arthritis and persistent neurological complications^2,9^.

In addition to *B. burgdorferi*, other pathogenic members of the *Borrelia* genus cause Lyme disease in Eurasia, including *B. garinii* and *B. afzelii*^8^. While the clinical syndromes overlap, there are species-specific differences in disease presentation. *B. burgdorferi* is more often associated with arthritis, *B. garinii* is linked primarily to neurological involvement, and *B. afzelii* is most frequently connected to the dermatologic condition acrodermatitis chronica atrophicans^3,8^.

Lyme disease is a complex infection that can progress through multiple stages and affect various organ systems. Among its neurological complications, collectively termed Lyme neuroborreliosis, the most frequent are painful meningoradiculitis (Bannwarth syndrome) and lymphocytic meningitis^9^. In patients with Lyme neuroborreliosis, antibiotic therapy has been shown to reduce disease duration and the likelihood of neurological sequelae; however, the most appropriate antibiotic selection and treatment duration continue to be matters of clinical uncertainty. Treatment for Lyme neuroborreliosis typically involves either oral doxycycline or intravenous ceftriaxone^10–12^. Doxycycline, while convenient and orally administered, is bacteriostatic and may not fully eliminate *B. burgdorferi* in all cases. Ceftriaxone is bactericidal and more effective for neuroborreliosis but requires intravenous administration, making it less practical for long-term use^11^.

Although generally effective, these antibiotics have drawbacks since broad-spectrum compounds disrupt the gut microbiome and promote resistance in off-target bacteria^13–15^. The intestinal microbiome plays a central role in immune development, maintenance of gastrointestinal health, and protection against cardiovascular, neurological, and autoimmune disease^13,14^. For these reasons, the development of narrow-spectrum antibiotics that act selectively against *B. burgdorferi* is a desirable therapeutic goal.

Given the continued rise in Lyme disease cases, the absence of a reliable vaccine^16^, and the limitations of current antimicrobial regimens^12^, there is a pressing need for innovative therapies. Targeted antibiotics represent a promising strategy to improve outcomes for patients with both early and disseminated Lyme disease and to lessen the long-term public health burden of this increasingly common infection.

We previously reported that hygromycin A, a protein synthesis inhibitor, is selective against spirochaetes and shows efficacy in a mouse model of Lyme disease^17^. However, hygromycin A has limited oral bioavailability. In search of additional promising compounds, we found that forazemin (formerly BB-83698) is highly active against spirochaetes and largely spares microbiome commensals. It inhibits peptide deformylase (PDF), an essential enzyme that catalyzes the removal of formyl groups from nascent bacterial proteins^18^. In mouse infection models, oral administration of forazemin effectively eradicated *B. burgdorferi* while causing minimal disruption of the gut microbiome. The selective antibacterial activity of forazemin, together with the essential role of its peptide deformylase target and the limited potential for resistance emergence, indicates that forazemin could be useful candidate for the treatment and prevention of Lyme disease.

### Identification of forazemin as a selective compound acting against *B. burgdorferi*

**Figure 1:**
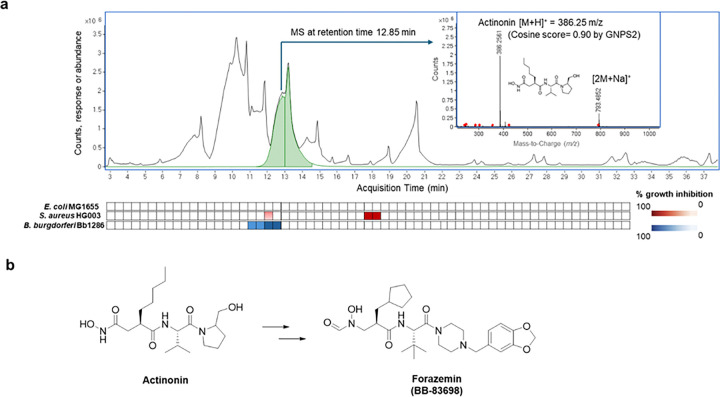
Identification of a compound from a selective screen against *B. burgdorferi*. **a.** Bioassay-guided metabolomics LCMS/MS identification of secondary metabolites from *Streptomyces sp.* (ATCC 14903) active against *B. burgdorferi*. The counter screen was performed against *S. aureus* and *E. coli*. Darker color means more inhibition compared with control growth. Mass spectra of dominant *B. burgdorferi* selective metabolite, exact mass (M+H)^+^ 386.25 *m/z*; dereplicated to actinonin by Global Natural Product Social Molecular Networking (GNPS2). **b.** Structures of actinonin and the synthetic PDF inhibitor developed by British Biotech BB-83698 renamed as forazemin.

Many of the antibiotics in current use trace their origins to soil actinomycetes. Although extensively investigated, prior discovery programs largely focused on broad-spectrum agents. In search of compounds acting selectively against spirochaetes, we carried out differential screening of *Streptomyces* extracts against *B. burgdorferi*, using *S. aureus* as a counter-screen. This identified actinonin from *Streptomyces sp.* (ATCC 14903) (Fig. 1a) with preferential activity against *B. burgdorferi* (Table 1). Actinonin inhibits bacterial PDF, and is most active against fastidious Gram-negative species, *Haemophilus and Moraxella* (MIC ~0.5–2 μg ml^−1^); it is fairly inactive against *Escherichia* and *Pseudomonas* with a restrictive penetration barrier (MIC > 64 μg ml^−1^), and has modest activity against Gram-positive species, *Staphylococcus* (MIC ~ 16–32 μg ml^−1^) and *Bacillus* (MIC 4–8 μg ml^−1^)^19^. Development of actinonin was precluded due to toxicity resulting from its inhibition of mitochondrial PDF^20^. Efforts to design non-toxic inhibitors have focused on exploiting structural differences between bacterial and human mitochondrial PDF, achieving >1000-fold selectivity^21^. We tested available PDF inhibitors against *B. burgdorferi* and non-target bacteria (*S. aureus* and *E. coli*) to evaluate selectivity. Cytotoxicity assays were performed in parallel (Supplementary Table 1). This initial screen showed that the selectivity window between *B. burgdorferi* and *S. aureus*/*E. coli* was the largest in case of BB-83698, a synthetic analog of actinonin, which we renamed forazemin (Fig. 1b).

### Forazemin is active against spirochaetes

We assessed the activity of forazemin against two families of pathogenic Spirochaetales: Spirochaetaceae and Leptospiraceae (Table 1, Extended Data Table 1). Forazemin displayed potent activity across both families. It showed the greatest activity against *Borrelia hermsii* (MIC 0.125 μg ml^−1^), the primary etiological agent of tick-borne relapsing fever. Forazemin was active against *B. burgdorferi* (MIC 0.5–1 μg ml^−1^), the leading cause of Lyme disease in the US; *Borrelia miyamotoi* (MIC 0.5 μg ml^−1^), an emerging relapsing fever agent transmitted by *Ixodes* ticks across North America, Europe, and Asia^8^, causing febrile illness with neurological complications. It showed potent activity against *Borrelia afzelii* (MIC 0.5 μg ml^−1^), a primary cause of skin manifestations of Lyme borreliosis and acrodermatitis chronica atrophicans in Europe, and *Borrelia spielmanii* (MIC 1 μg ml^−1^), a rodent-associated European Lyme agent linked to *Ixodes ricinus* ticks that infects humans, forming erythema migrans. Forazemin was active against diverse *Borrelia* isolates worldwide, transmitted by distinct tick vectors (Extended DataTable 1). Activity extended to *Treponema pallidum* (MIC 0.125 μg ml^−1^), the causative agent of syphilis^22^. Forazemin was also highly effective against Leptospiraceae *Leptospira biflexa.*

Forazemin is relatively ineffective against both Gram-positive and Gram-negative gut symbionts tested so far (Table 1, Supplementary Table 2). Forazemin lacks activity against Enterobacteriaceae such as *E. coli*, non-fermentative Gram-negative bacilli *P. aeruginosa*, and non-spore forming fermentative Gram-positive lactobacilli and bifidobacteria. Forazemin is bacteriostatic against most Gram-positive pathogens with the exception of *Streptococcus pneumoniae*^23^ but proved bactericidal against *B. burgdorferi*, with a minimum bactericidal concentration (MBC) of 2 μg ml^−1^ (at 4×MIC) while doxycycline is largely bacteriostatic with an MBC of 64 μg ml^−1^ (Extended Data Fig.1).

High frequency of resistance development is a general liability of PDF inhibitors^18,24^. Notably, plating a high-density *B. burgdorferi* culture (~10^9^ CFU) onto agar containing forazemin at 4x, 8x and 16x MIC produced no resistant mutants indicating a mutation frequency below 10^−9^.This finding suggested further evaluation of forazemin as a potential therapeutic for Lyme disease.

### Forazemin inhibits peptide deformylase in *B. burgdorferi*

Forazemin acts by specifically inhibiting PDF, a bacterial metalloenzyme. While iron (Fe^2+^) is the typical PDF cofactor in many bacteria such as *Escherichia coli*, zinc (Zn^2+^) likely serves as the cofactor in *Borreliella* and *Leptospira*^25^. Molecular docking was performed using Maestro by Schrödinger (version 2025–4) to assess the binding affinity of forazemin to PDF of *B. burgdorferi* (BbPDF). The compound was first docked into the PDF of *L. interrogans* (LiPDF) for which a crystal structure in complex with actinonin is available (PDB: 1SZZ). Actinonin was then replaced by forazemin in the active site. For BbPDF, the absence of an available crystal structure necessitated generation of a co-folded structural model incorporating actinonin, Zn^2+^, and BbPDF using the Boltz-2 algorithm (Supplementary Table 3). Comparative docking analysis revealed tight binding of forazemin with both BbPDF and LiPDF. Examination of the forazemin-BbPDF binding mode shows that the compound establishes a five-membered bidentate coordination complex with the catalytic zinc ion through interactions involving Zn^2+^, enzyme residues H130, H134, E131, C88, and the hydroxamate functional group of forazemin. The hydroxamate moiety concurrently forms a trifurcated hydrogen bonding network with Q48 and H131, providing additional binding stabilization. The peptidic portion of forazemin engages in backbone interactions with V42, G87, and E86 within the peptide recognition domain of BbPDF. The benzo[1,3]dioxole substituent occupies a hydrophobic binding pocket adjacent to Y84 at the interface between the N-terminal and C-terminal domains, contributing favorable hydrophobic interactions to the overall binding energy.

**Figure 2:**
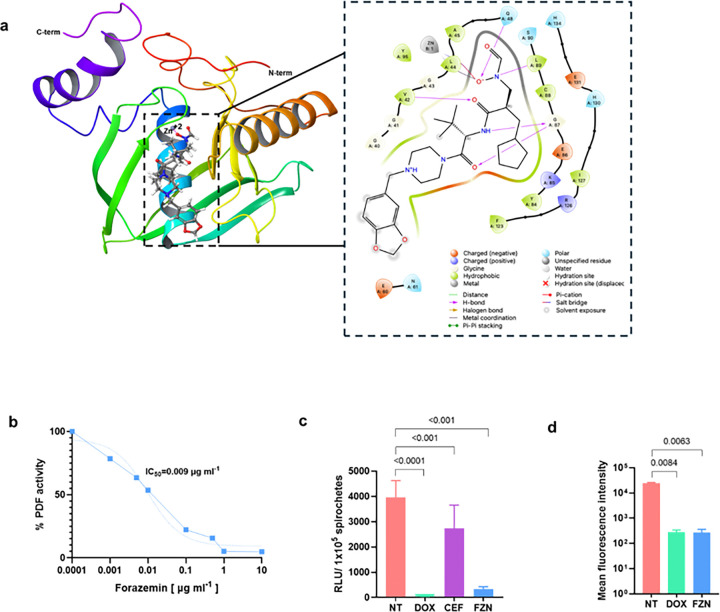
Mechanism of action of forazemin against *B. burgdorferi*. **a.** Forazemin bound to BbPDF (Boltz-2) located in the metallo-catalytic pocket centered between the N- and C-terminal sub-domains and a 2D scheme of forazemin binding profile at 4. **b.** Dose-response curves of BbPDF enzyme inhibition by forazemin. N-formylmethionyl-leucyl-*p*-nitroanilide (f-ML-*p*NA) was used as a substrate, and the peptide deformylase (PDF) reaction is coupled with *Aeromonas* aminopeptidase (AAP). Deformylation by PDF releases ML-*p*-nitroanilide, which is sequentially hydrolyzed by AAP to release the chromophore *p*-nitroaniline (absorbance at 405 nm , A_405_). PDF activity was expressed as the rate of *p*-nitroaniline released from the peptide (*n* = 3 experiments). **c.** Protein synthesis assay. *B. burgdorferi* expressing firefly luciferase under IPTG induction. Bacteria were treated with 2xMIC of antibiotics (forazemin FZN, ceftriaxone CEF or doxycycline DOX). Luciferase activity was measured with D-luciferin and normalized by 10^5^ cell counts. Data represent mean ± s.e.m. from *n* = 5 biological experiments. Statistical analysis was performed by one-way ANOVA with Dunnetťs multiple comparisons test (each treatment versus non-treated control). *P* values are shown above datasets. **d.** Nascent proteome synthesis assay by non-canonical amino acid incorporation. The incorporation of β-ethynylserine (βES), analog of threonine into nascent protein synthesis was observed with 5-TAMRA azide by click chemistry. Bacteria were treated with 2xMIC of antibiotics (forazemin or doxycycline). Data represent mean ± s.e.m. Statistical significance was determined by one-way ANOVA with Tukey's multiple comparisons test. *P* values are shown above datasets.

To confirm the target of forazemin, we tested its ability to inhibit deformylation of a substrate by purified recombinant BbPDF. Forazemin inhibited peptide deformylase in a dose-dependent manner with very low inhibitory concentration (IC_50_) of 9 ng ml^−1^ (Fig. 2b). To confirm the effect of forazemin on protein synthesis, we used a strain of *B. burgdorferi* that ectopically expresses firefly luciferase. Cells were treated with either forazemin or doxycycline, a known protein synthesis inhibitor, and ceftriaxone, a cell wall synthesis inhibitor, and luciferase expression was induced with isopropyl β-D-1-thiogalactopyranoside (IPTG). Forazemin strongly inhibited expression of luciferase, comparable to that of doxycycline. In contrast, ceftriaxone had no significant effect on luciferase production, confirming that forazemin specifically inhibits protein synthesis (Fig. 2c). To observe the effect of forazemin on the nascent proteome, we utilized bioorthogonal non-canonical amino acid tagging using the threonine analog β-ethynylserine (βES) for tracking protein synthesis. Forazemin blocked the incorporation of β-ethynylserine, indicating an inhibition of nascent protein synthesis (Fig. 2d). Since forazemin inhibits deformylation of nascent peptides, we sought to determine if this would result in accumulation of formylated proteins. Protein lysates from *B. burgdorferi* cultures treated with forazemin were analyzed by SDS-PAGE and western blotting for formylated proteins (Supplementary Fig. 1). Forazemin-treated lysates showed a prominent band between 35 and 55 kDa whose intensity was markedly increased compared with the untreated control indicating that BbPDF inhibition leads to accumulation of formylated proteins in *B. burgdorferi*. Scanning electron microscopy and epifluorescence microscopy showed pronounced morphological defects, including surface blebbing and loss of the characteristic spiral shape (Supplementary Fig. 2 and 3). No pleomorphic or cyst-like forms were observed, unlike those induced by β-lactam antibiotics. These results demonstrate that forazemin inhibits peptide deformylase, leading to accumulation of N-formylated proteins and cell death.

### Forazemin is efficacious in murine models of Lyme borreliosis and neuroborreliosis.

Given the high in vitro potency and selectivity of forazemin against *Borrelia*, we evaluated its potential as a therapeutic agent. Testing forazemin against human cell lines showed no cytotoxicity (CC_50_ >512 μg ml^−1^), giving remarkable in vitro therapeutic index of >1,000 (Supplementary Table 4). Oral antibiotics represent first-line therapy for Lyme disease. Although the Phase I trial evaluated an intravenous formulation of forazemin, mouse studies indicated 50% oral bioavailability for the compound^26^. To confirm this finding, we performed single-dose pharmacokinetics with forazemin administered orally to mice at 50 mg kg^−1^. Forazemin showed favorable C_max_ of 4.5 μg ml^−1^ and half-life T_1/2_ of ~1 h (Extended Data Fig. 2). To evaluate blood-brain barrier (BBB) penetration, we performed hCMEC/D3 Transwell assays; forazemin crossed the monolayer at rates comparable to ceftriaxone/doxycycline, the standard for neuroborreliosis (Extended Data Fig. 3). These findings prompted oral efficacy testing in murine models of Lyme borreliosis and Lyme neuroborreliosis.

**Figure 3:**
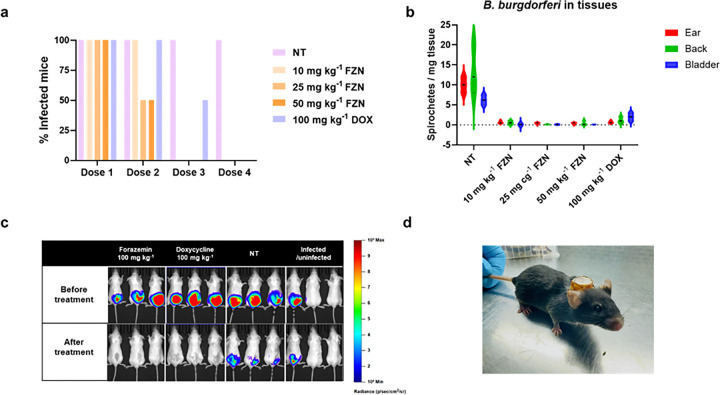
Efficacy of forazemin in a murine Lyme borreliosis model. **a.** C3H/HeN mice were infected subcutaneously with *B. burgdorferi* N40, and after 3 weeks were treated for 5 days with forazemin (FZN) or doxycycline (DOX) via oral gavage. All mice were treated twice a day, and the total daily dose (mg kg^−1^) is indicated. After treatment, the presence of *B. burgdorferi* cells was detected by dark-field microscopy from culture of a whole ear in BSK-II media. The percentage of mice from which cultures were positive is reported. **b.** Quantification of *B. burgdorferi* in tissues (ear, back and bladder) of animals. The borrelia burden was quantified by dark-field microscopy and normalized by amount of tissue in mg. Five mice per group were infected and given forazemin (FZN) or doxycycline (DOX) orally; infected non-treated group (NT) was given drug-free vehicle for 5 days. **c.** Mice were infected with *B. burgdorferi* ML23 pBBE22luc (P_*flaB*_-*luc*) and randomized to receive either forazemin or doxycycline orally. Mice were injected intraperitoneally with D-luciferin and imaged at 1 h; representative images derive from independent infections. All images for a given time point and strain were normalized to the same photon flux range and color scale. **d.** Murine prophylaxis model. C3H/HeNCrl mouse carrying cap containing five nymphal *Ixodes scapularis* to mimic tick bite.

We initially assessed the efficacy of forazemin in a murine model of Lyme borreliosis. C3H/HeN mice were subcutaneously infected with *B. burgdorferi* N40 and allowed to develop a disseminated infection over a 3-week period^17^. Forazemin was subsequently administered orally by gavage at doses of 10, 25, or 50 mg kg^−1^ twice daily for 5 consecutive days. Bacterial burden was quantified by culture positivity of organ biopsies in liquid medium and by 16S rRNA quantitative PCR^17^. Because a single viable spirochete can generate detectable growth in liquid culture, this method provides a stringent measure of therapeutic efficacy. Forazemin achieved complete clearance of infection across all treatment groups, comparable to doxycycline. All mice across all dose groups were culture-negative after four doses, surpassing the efficacy of the high dose doxycycline control group (Fig. 3a). Furthermore, 50% of mice treated with 25 or 50 mg kg^−1^ achieved clearance after just two doses, indicating 2- to 4-fold greater potency relative to doxycycline. Mice that were culture-negative exhibited no bacterial rebound in ear tissue through day 12 following treatment initiation, and no spirochaetes were detected in ear, back skin or bladder samples (Fig. 3b).

The use of intradermal inoculation recapitulates the natural route of infection via tick bite; we additionally evaluated the therapeutic efficacy of forazemin in a bioluminescent *B. burgdorferi* murine infection model. To compare its performance with a standard antibiotic, high-dose regimens of forazemin (100 mg kg^−1^, twice daily) and doxycycline (100 mg kg^−1^, twice daily) were administered to mice infected intradermally with the bioluminescent *B. burgdorferi* ML23 pBBE22luc (P_*flaB*_-*luc*), which permits real-time, spatiotemporal monitoring of bacterial dissemination^27^. A single high-dose treatment with forazemin achieved infection clearance comparable to that observed with doxycycline (Fig. 3c).

We next assessed the efficacy of forazemin in a murine model of neuroborreliosis, in which *B. burgdorferi* colonizes the dura mater. C3HeB/FeJ mice were infected intradermally with *B. burgdorferi* B31-A3, and spirochaetes were allowed to disseminate seven days post-infection^28^. Animals were then treated orally with forazemin or doxycycline (100 mg kg^−1^ day^−1^) for five consecutive days. Consistent with prior observations, *B. burgdorferi* was not detected in the brain tissue of infected animals by culture, irrespective of treatment. In contrast, dura mater samples from untreated mice remained culture-positive up to 75 days post-infection. As expected, untreated controls yielded viable spirochaetes, whereas no *B. burgdorferi* were recovered from the dura mater of mice treated with either forazemin or doxycycline (Table 2).

Doxycycline is the standard prophylactic for adults to prevent Lyme disease after a tick bite^29^. To test if forazemin can be used as prophylaxis, we compared single-dose efficacy in a murine model of tick-transmitted *B. burgdorferi* infection. Groups of C3H/HeNCrl mice received a single oral dose of forazemin or doxycycline (each at 100 mg kg^−1^) or vehicle control immediately following removal of *Ixodes scapularis* nymphs infected with *B. burgdorferi* (Fig. 3d). Three weeks later, tissue cultures revealed that forazemin protected 1 of 4 mice (25%) when administered immediately, and 2 of 4 mice (50%) at 48 h and 96 h, while doxycycline offered no protection under the same conditions. We subsequently evaluated a two-dose regimen with C3H/HeN mice exposed to infected ticks (two oral doses of forazemin or doxycycline, 100 mg kg^−1^, within 24 h of tick removal). Forazemin fully protected all animals from infection, as confirmed by sterile cultures, while doxycycline again failed to prevent borreliosis (Table 3).

### Forazemin spares the symbiotic microbiome

**Figure 4:**
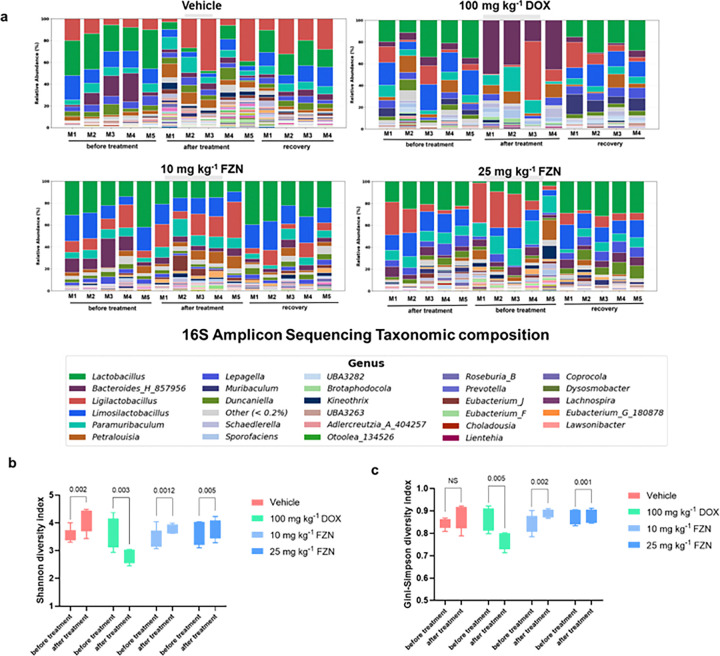
Effect of forazemin on murine microbiome. **a.** The change in relative abundance of the most abundant genera in the murine gut microbiome following treatment with doxycycline (DOX) and forazemin (FZN). Four to five mice per treatment group were infected with *B. burgdorferi* N40 and treated twice a day for 5 days; the control received vehicle. Stool was collected before and after treatment and recovery, and 16S rRNA sequencing was used to bacterial community composition. Taxonomic relative abundance at genus level of fecal samples before and after, recovery for vehicle, 100 mg kg^−1^ doxycycline, 10 mg kg^−^1 and 25 mg kg^−1^ forazemin. Each point represents the change in the relative abundance of the respective genus for an individual mouse across three individual experiments. **b.** The change in alpha diversity based on the Shannon index of the murine fecal microbiome following treatment with doxycycline (DOX) and forazemin (FZN) administered orally. Alpha diversity was calculated using the Shannon Index at before treatment (day 0) and after treatment (day 5). For the box-and-whisker plot: box limits, 25th and 75th quartiles; whiskers, minimum and maximum values. Statistical significance was calculated using a one-way ANOVA with Tukey’s multiple comparisons test. **c.** Simpson diversity index of the fecal microbiota before and after vehicle, doxycycline (DOX) and forazemin (FZN) treatment. Alpha diversity was assessed using the Gini-Simpson index (1-λ) in fecal samples collected at before treatment (day 0) and after treatment (day 5). A higher Simpson index reflects greater species evenness and diversity. Statistical significance was determined by a one-way ANOVA with Tukey’s multiple comparisons test.

Given the in vitro specificity and low-dose efficacy of forazemin against *B. burgdorferi* in murine infection models, we next investigated its impact on the gut microbiome at efficacious doses and compared that to doxycycline. Mice infected with *B. burgdorferi* N40 received clinically relevant oral doses of forazemin (10 or 25 mg kg^−1^ for therapeutic evaluation; 100 mg kg^−1^ for prophylactic dosing), doxycycline (100 mg kg^−1^), or vehicle control. Gut microbial composition was characterized by 16S rDNA sequencing of fecal samples. The baseline murine gut microbiota was dominated by members of the phyla Bacteroidota (notably Muribaculaceae, including Paramuribaculum), Firmicutes (including *Lactobacillus* spp.), and Actinobacteriota. Doxycycline has a pronounced effect on murine gut^30^. We found that doxycycline administration induced pronounced dysbiosis whereas forazemin treatment promoted enrichment of commensal Firmicutes - particularly lactate-producing *Lactobacillus* spp. consistent with in vitro susceptibility and elevated levels of health-associated Muribaculaceae taxa (Fig. 4a). The Shannon alpha diversity of doxycycline-treated mice was significantly altered, whereas Shannon diversity in forazemin-treated mice did not change significantly and remained comparable to that of untreated controls, indicating minimal impact on the microbiome (Fig. 4b). Treatment with forazemin did not significantly alter the Simpson diversity index relative to vehicle, in contrast to doxycycline, which produced a marked reduction in gut microbial evenness, indicating a microbiome-sparing profile for forazemin. Following treatment cessation, the microbiome of forazemin-treated mice returned to baseline composition, in contrast to the persistent disruption observed in doxycycline-treated groups. Combined Lyme infection and broad-spectrum antibiotics often deplete key short-chain fatty acid (SCFA) -producing bacteria^31^. These community shifts corresponded with SCFA profiles: doxycycline suppressed SCFA-producing taxa (e.g., Muribaculaceae) and reduced total SCFA concentrations by more than 30% during treatment, while forazemin preserved both producer abundance and overall SCFA levels (Extended Data Fig. 4). Consistent with these findings, analysis of Shannon diversity indices revealed that forazemin maintained after treatment microbiome diversity comparable to before treatment levels, whereas doxycycline caused a pronounced decline with incomplete recovery after drug withdrawal. To assess whether forazemin affects murine microbiome during prophylaxis, C3H/HeN mice (n = 3 per group) received oral doses of 100 mg kg^−1^. 16S rRNA sequencing of fecal samples showed no significant changes in community composition after treatment (Extended Data Fig. 5). Both a single 100 mg kg^−1^ dose and two-dose regimen of 100 mg kg^−1^ preserved overall microbiome structure. These results suggest that forazemin, even at higher prophylactic doses, does not appreciably disturb microbiota and has a favorable selectivity profile towards *B. burgdorferi.* These data demonstrate that forazemin has a minimal effect on the murine gut microbiome while maintaining potent anti- *Borrelia* activity.

## Discussion

The rise in antibiotic resistance and the important role of the microbiome in human health support a shift from traditional broad-spectrum antibiotics toward selective or narrow-spectrum agents. Here, we report the identification of actinonin as an anti-Borrelia compound through differential screening, which led us to forazemin, a pharmacologically optimized analog with improved tolerability relative to actinonin. Actinonin is a non-selective inhibitor of peptide deformylase and is associated with toxicity where forazemin has no effect on human peptide deformylase. Though actinonin was abandoned due to unfavorable toxicity, BB-83698/forazemin successfully passed a Phase I clinical trial^26^. Its intended use against *S. pneumoniae* had limited marketing appeal, which precluded further development^23^. Historically, this class of antibiotics has had limited clinical use because of resistance emergence in target pathogens^18^. *B. burgdorferi* possesses a highly compact chromosome of approximately 910 kb, with a total genome including plasmids of ~1.5 Mb^32^, lacking many redundant metabolic and protein processing pathways found in other bacteria. This compact and reduced genome suggests limited alternative mechanisms for bypassing PDF inhibition, which may in turn reduce the likelihood of resistance development. Forazemin has several attractive properties, including good oral bioavailability, potent in vivo efficacy against *B. burgdorferi* at an effective dose of 10 mg kg^−1^, and minimal microbiome disruption.

Forazemin exhibits several properties that may offer advantages over current Lyme disease therapies. Tetracyclines can severely disrupt the human microbiome^14^. Similarly, doxycycline treatment for Lyme disease has been associated with a marked decline in key SCFA-producing commensal bacteria in macaques^33^. This reduction likely weakens the integrity of the gut barrier, resulting in heightened immune activation and systemic inflammation^33^. Doxycycline use expands tetracycline resistance genes in the gut microbiome^30^, which can then transfer into pathogens. Ceftriaxone is administered intravenously, but 30–40% is concentrated in bile and subsequently excreted into the gut, where it disrupts the microbiome^34,35^. By contrast, forazemin had little overall effect on the mouse microbiome, while preferentially enriching SCFA-producing taxa including *Lactobacillus* spp.

Forazemin is efficacious against both disseminated *B. burgdorferi* infection and neuroborreliosis in mice via oral dosing. Forazemin achieved blood-brain barrier penetration on par with established standard-of-care therapies. Forazemin may be an alternative to doxycycline or ceftriaxone for neuroborreliosis affecting the central nervous system. Unlike the bacteriostatic doxycycline, forazemin displayed cidality against spirochaetes. Ceftriaxone is bactericidal but needs to be administered intravenously^11^. These results suggest forazemin could be an oral alternative to current neuroborreliosis treatments, particularly given its bactericidal activity and CNS penetration demonstrated in the in vitro model. Oral bioavailability is especially valuable for antibiotics requiring prolonged courses: acute Lyme demands 10–14 days of doxycycline while delayed-diagnosis Lyme arthritis requires up to 3 months^36^. The favorable prophylactic efficacy and selectivity profile of forazemin suggest it could be explored as an alternative post-exposure prophylaxis strategy in future clinical studies, with adherence to multi-dose oral regimens potentially offering a practical advantage over existing approaches. Beyond Lyme disease, the broader activity of forazemin across the Spirochaetales order raises the possibility of therapeutic utility against other Spirochaetal pathogens, including *Treponema pallidum*, which causes syphilis, a sexually transmitted infection. Penicillin treats *T. pallidum* effectively but is limited by shortages, painful injections, and 10–20% early syphilis failure rates^22,37,38^. Patients often fail to complete doxycycline courses, and azithromycin has lost efficacy due to resistance, leaving limited treatment alternatives^37^. Forazemin potently inhibits *Treponema pallidum*, making it a candidate for evaluation in a rabbit efficacy model.

Repurposing a clinically evaluated antibiotic for activity against a new pathogen (*Borrelia*) is uncommon but holds significant therapeutic potential. With its demonstrated efficacy in murine models and minimal impact on the host microbiota, forazemin represents a promising clinical candidate for the development of a targeted therapy against Lyme disease.

## Methods

### Bacterial Strains

Strains were obtained from either the American Type Culture Collection (ATCC) or Deutsche Sammlung von Mikroorganismen und Zellkulturen (DSMZ). Anaerobic bacteria were isolated from fecal samples collected from healthy human donors under an Institutional Review Board (IRB)-approved protocol (IRB # 24–11-13 at Northeastern University). *Streptomyces* sp. ATCC 14903 was maintained on GT agar (soluble starch 20 g l^−1^, L-asparagine 0.5 g l^−1^, KNO_3_ 1.0 g l^−1^, K_2_HPO_4_·H_2_O 0.5 g l^−1^, NaCl 0.5 g l^−1^ , MgSO_4_·7H_2_O 0.5 g l^−1^; pH 7.5) until sporulation, and spore stocks were stored at −80 °C. All *Borrelia* strains, including *B. burgdorferi* B31, BbP1286, N40 and 297, as well as *B. afzelii*, *B. garinii*, *B. bavariensis*, *B. miyamotoi* and *B. andersonii*, were cultured in Barbour-Stoenner-Kelly II (BSK-II) medium at 34 °C under microaerophilic conditions (3% O_2_, 5% CO_2_)^17,39^. For *B. burgdorferi* BbP1286, which constitutively expresses GFP under control of the flaB promoter, cultures were supplemented with 50 μg ml^−1^ gentamicin and 100 μg ml^−1^ kanamycin^40^. *B. burgdorferi* ML23 pBBE22luc, a clonal B31 derivative lacking lp25 and carrying bbe22 and a codon-optimized luciferase gene under the *flaB* promoter (P*flaB*-luc), was used for in vivo bioluminescence imaging^27^. VivoGlo D-luciferin (Promega) was used as the substrate for luminescence detection.

*Brachyspira hyodysenteriae* B78 (ATCC 27164) was grown anaerobically in brain heart infusion broth supplemented with 10% (v/v) fetal calf serum at 37 °C for 48 h to mid-logarithmic phase before use in susceptibility testing. *Leptospira biflexa* Patoc 1 and *L. interrogans* serovar Copenhageni were cultured statically at 30 °C in Ellinghausen-McCullough-Johnson-Harris (EMJH) medium.

*T. pallidum* was cultivated in a coculture system with Sf1Ep rabbit epithelial cells^41^. Sf1Ep cells were seeded in 24-well plates and allowed to adhere overnight at 37 °C in 5% CO_2_. Before inoculation, cells were washed with TpCM2 and equilibrated at 34 °C under microaerophilic conditions (1.5% O_2_, 5% CO_2_) for at least 3 h. *T. pallidum* harvested from existing cocultures was added at approximately 2.5 ×10^6^ CFU ml^−1^ per well in 2.5 ml TpCM2. Cocultures were maintained at 34 °C in an atmosphere of 1.5% O_2_ and 5% CO_2_ for 8 d to support treponemal growth.

Broth microdilution MICs for *E. coli* MG1655 and *S. aureus* HG003 used Mueller Hinton II broth (CAMHB) with 5×10^5^ CFU ml^−1^ inoculum from mid-log cultures in 96-well plates, incubated aerobically at 37°C for 18 h; MIC was the lowest concentration with no visible growth^42^. A suspension of the anaerobic bacterium was prepared in ATCC Medium 1293 (BHI broth supplemented with hemin and vitamin K1) and inoculated into two-fold serial dilutions of antibiotics in the same medium to achieve a final inoculum of 5 × 10^5^ CFU ml^−1^.

### Cytotoxicity assay

Primary human lung fibroblasts and mammalian cell lines-human alveolar basal epithelial (A549), human Caucasian colon adenocarcinoma (Caco-2), FaDu pharyngeal squamous cell carcinoma (ATCC HTB-43), HepG2 hepatocellular carcinoma (ATCC HB-8065), and human embryonic kidney (HEK293) were obtained from the American Type Culture Collection (ATCC) or equivalent sources and cultured in Eagle’s minimum essential medium supplemented with 10% fetal bovine serum at 37 °C, 5% CO_2_. Cells were seeded into 96-well plates, allowed to adhere for 24 h, then treated with two-fold serial dilutions of forazemin for 72 h. Cytotoxicity was assessed using the Alamar Blue assay (performed in triplicate): 3 mM resazurin was added for 3 h, followed by fluorescence measurement (excitation 544 nm, emission 590 nm) on a microplate reader. DMSO (1:1 v/v in growth medium) served as the positive cytotoxicity control; untreated cells were the viability control.

### Antibacterial assays

Microbroth dilution minimum inhibitory concentrations (MIC)s were determined for forazemin against *Borrelia*, *Leptospira*, *Treponema*, aerobic lab strains (like *S. aureus* HG003, *E. coli* MG1655), pathobionts and symbionts (all ≥3 biological replicates). Exponential phase cultures were diluted 1:10 into 96-well plates containing two-fold serial dilutions of forazemin (1:100 for *L. biflexa*); growth controls were included. *Borrelia* cultures were incubated microaerophilically (34 °C, 3% O_2_, 5% CO_2_, 7 d; phenol red color change scored visually) and confirmed by dark-field microscopy spirochete counts. *Leptospira* was grown aerobically (6 d; resazurin 0.001 g l^−1^ color change scored). *T. pallidum* was tested in Sf1Ep co-culture: 6-well plates (pre-equilibrated TpCM-2) were inoculated with 3.3–3.5 × 10^6^ trypsinized spirochaetes per well, incubated low-oxygen (1.5% O_2_, 5% CO_2_, 93.5% N_2_; 7 d at 34 °C), trypsinized, quantified by dark-field microscopy (≥2 counts per Helber chamber; motility scored), and MIC interpolated as inoculum-equivalent yield. Aerobic strains started at 5 × 10^5^ CFU ml^−1^ and were incubated 16–20 h (37 °C); anaerobes were diluted 1:100 and incubated 24–48 h anaerobically. MICs were scored visually as the lowest concentration preventing growth. For minimum bactericidal concentration (MBC), exponential phase *B. burgdorferi* B31 cells (10^7^ CFU ml^−1^) were incubated with forazemin at 1×, 2×, 4× and 8× MIC in 1.5-ml tubes for 5 d (microaerophilic, 34 °C). Cells were washed in BSK-II, serially diluted onto semi-solid BSK-II plates, and incubated microaerophilically for 20 d. Minimum Bactericidal Concentration (MBC) was scored as the lowest concentration reducing viability by >3 log_10_ CFU. For spontaneous resistant mutants generation, *B. burgdorferi* B31 cells (10^9^ CFU ml^−1^) were plated on semi-solid BSK-II plates and incubated microaerophilically for 30 d. For time-kill kinetics, exponential phase *B. burgdorferi* B31 cells (10^7^ CFU ml^−1^) were treated with either forazemin or doxycycline (10xMIC), and at predefined time points (0, 12, 24, 48 and 72 h), 0.5 ml samples were quantified by colony forming units on BSK-II agar plates.

### Anti-*Borrelia* compound screening

Bacterial strains like *Streptomyces* were grown as mentioned above^17^. Anti-*Borrelia* activity was tested against GFP-expressing *B. burgdorferi* BbP1286 (grown microaerophilically in BSK-II + 50 μg ml^−1^ gentamicin, 100 μg ml^−1^ kanamycin); we diluted exponential-phase culture 1:100 and added 197 μl to 3 μl extract in 96-well plates (7 d, 34 °C, 3% O_2_, 5% CO_2_), scoring ≥80% GFP fluorescence reduction (excitation 485 nm, emission 528 nm; Synergy H1 reader) versus extract-free controls as positive. We counter-screened 3 μl extract diluted into 97 μl CAMHB with *S. aureus* HG003 or *E. coli* MG1655 (OD_600_ = 0.001); growth inhibition indicated anti-staphylococcal or anti-*E. coli* activity relative to controls.

### Dereplication of *Borrelia* active compound

Compounds selectively active against target pathogens were rapidly dereplicated using a high-throughput screening approach that integrates liquid chromatography–tandem mass spectrometry (LC-MS/MS) with a bioactivity-guided fractionation assay^42^. *Streptomyces* sp. ATCC 14903 seed cultures (5 d, ISP-2 medium: 4 g l^−1^ yeast extract, 10 g l^−1^ malt extract, 4 g l^−1^ dextrose) were inoculated into 250-ml MPG medium (10 g l^−1^ dextrose, 20 g l^−1^ millet meal, 20 g l^−1^ cottonseed gluten meal, 20 g l^−1^ MOPS; pH 7.2) in 1-l baffled flasks (28 °C, 150 r.p.m., 7 d)^43^. Supernatants were extracted twice with ethyl acetate (1:1 v/v), evaporated to dryness. Ethyl acetate extracts of producer culture supernatants were fractionated using an Agilent 1260 Infinity LC system coupled to an Agilent 6530 quadrupole time-of-flight (QTOF) mass spectrometer equipped with an electrospray ionization (ESI) source (Agilent Technologies). A post-column flow splitter diverted the eluent simultaneously to a fraction collector equipped with a 96-well microtiter plate and to the mass spectrometer. Chromatographic separation was performed on a Waters XBridge C18 column (250 mm × 4.6 mm, 5 μm) at a flow rate of 1 ml min^−1^ using a binary solvent system consisting of solvent A (0.1% formic acid in water, v/v) and solvent B (0.1% formic acid in acetonitrile, v/v). The gradient was initiated at 20% B, increased linearly to 100% B over 30 min, and held at 100% B for an additional 6 min.

MS data were acquired in positive ion mode using Auto MS/MS with the following parameters: gas temperature, 300 °C; drying gas flow, 7 L min^−1^; nebulizer pressure, 35 psi; fragmentor voltage, 175 V; skimmer voltage, 65 V. MS1 data were collected over 111–2000 *m/z* at 2 spectra s^−1^, and MS2 fragmentation spectra were acquired over 50–1500 *m/z* at 4 spectra s^−1^ with a collision energy of 10 eV, a maximum of 10 precursors per cycle, and active exclusion enabled after 5 spectra with a 0.5 min release window. Raw data were processed using MassHunter Qualitative Analysis software (Agilent Technologies) to extract putative masses from bioactive fractions. Fractions were tested against different pathogens for antimicrobial activity. Spectral library matching for the masses of interest identified from active fractions was performed using the librarysearch_workflow available on the GNPS2 platform (https://gnps2.org), with the following parameters: precursor mass tolerance, 0.05 Da; fragment mass tolerance, 0.05 Da; minimum matched peaks, 4; and minimum cosine similarity score, 0.7, queried against the GNPS spectral libraries.

### Computational Chemistry Methods

Maestro (version 2025–4) by Schrödinger was used to screen the small PDF inhibitor library. Briefly, the PDF library was prepared using LigPrep to create energy-minimized three-dimensional structures. The OPLS3e force field was used for minimization. Epik was used to generate all the possible ionized states, including metal binding sites at pH 7.0 ± 2.0. The desalt setting was used to remove any counter ions or water molecules. Tautomers and stereoisomers were generated (at most 32 per ligand) where specified chiralities were retained. For the LiPDF, SaPDF and EcPDF, in complex with Actinonin were taken from the PDB, (PDB: 1SZZ, 1LRU and 1Q1Y, respectively). For BbPDF, the FASTA for *def* (UNIPROT: O51092) was provided as the input sequence, alongside the co-factors of Actinonin and Zn^2+^ to generate three-dimensional structures in Boltz-2^44^. The resultant model selected for modelling was accurate with the following outputs, structure confidence: 0.97, Complex pLDDT: 0.96, ipTM:0.98, and pTM:0.97. The generated model had an RMSDs to the LiPDF system of 4.1Å. These structures were minimized (OPLS-20) using Protein Preparation Workflow, which assigned bond orders, added hydrogens, created zero-order bonds to metals, generated disulfide bonds, filled in missing side chains and loops using Prime, generated het states using Epik at pH 7.0 ± 2.0, and deleted water molecules beyond 5 Å from het groups. The resultant systems were used for docking studies using the Glide workflow, the grid was defined around Actinonin in each system. Next, the PDF library was docked at standard-precision (SP) to quickly assess the panel of inhibitors as binder to BbPDF, the resultant docking scores can be found in Supplementary Table 3.

### Peptide deformylase inhibitors screening

A synthetic library of PDF inhibitors was screened against *B. burgdorferi* BbP1286 using the same GFP fluorescence assay as for actinomycete extracts: 2 μl synthetic compound (10 mM DMSO stocks) was added to 198 μl diluted stationary-phase culture in 96-well plates (7 d microaerophilic incubation). Hits were defined as ≥80% GFP reduction versus vehicle controls and counter screened for selectivity (*S. aureus*, *E. coli*) and cytotoxicity against HEK293 cells (Alamar Blue assay)^17^.

### Peptide deformylase enzyme inhibition assay

Recombinant *Borrelia burgdorferi* B31 peptide deformylase (CSB-EP017707BUD; UniProt O51092) was purchased from CUSABIO. Assay reactions (total volume: 0.2 ml) were performed at room temperature in 50 mM potassium phosphate buffer (pH 7.0), supplemented with 10 mM NaCl, 1.0 mM tris(2-carboxyethyl) phosphine (TCEP), and 150 μM f-ML-pNA. *Borrelia burgdorferi* B31 peptide deformylase (BbPDF) Zn-PDF (20–256 ng ml^−1^) was added, along with varying concentrations (0.001–10 μg ml^−1^) of inhibitor forazemin. Reactions were incubated for 30 min at 37°C and terminated by heat inactivation at 95°C for 5 min. After cooling to room temperature, the reaction mixture was then incubated with 1 unit of *Aeromonas* aminopeptidase for 2–4 min. The release of *p*-nitroaniline was quantified by measuring absorbance at 405 nm^45^.

### Measurement of protein synthesis inhibition

Exponential cultures of the IPTG-inducible luciferase expressing *Borrelia burgdorferi* were treated with antibiotics at 2x MIC overnight (ceftriaxone 0.12 ug ml^−1^, doxycycline 0.5 ug ml^−1^, forazemin 1 μg ml^−1^). Cultures were then induced with 1 mM IPTG for 8 h. No-drug cultures and uninduced cultures were used as controls. 100 μl of bacterial culture were transferred to a white flat-bottom microtiter 96 well plate. Bioluminescence was evaluated from three independently grown cultures under the appropriate conditions and treatment. Luminescence was measured using plate reader (Synergy H1, BioTek Instruments). Each sample was treated with a final concentration of 667 μM D-luciferin (Sigma Aldrich) in PBS and after 30 min measured for luminescence. Each sample was measured for luminescence in triplicate; Cells were quantified using a BD FACS Aria II flow cytometer with a 70-μm nozzle. *B. burgdorferi* cells were gated by size using forward scatter (FSC) and side scatter (SSC). Relative luminescence from samples was normalized by number of spirochaetes.

### Nascent protein synthesis assay

The nascent protein synthesis rates were measured using the THRONCAT (threonine-derived non-canonical amino acid tagging) bioorthogonal metabolic labeling assay^46^. Cells were incubated with 4 mM of threonine analog β-ethynylserine (βES) for 24 h under standard culture conditions. Following incubation, 2 ml of each sample was collected and washed once with phosphate-buffered saline (PBS). Cells were fixed in 4% paraformaldehyde (PFA) for 20 min at room temperature, washed with PBS containing 3% bovine serum albumin (BSA), and subsequently permeabilized with 0.5% Triton X-100 in PBS for 20 min at room temperature. After permeabilization, cells were washed again with PBS containing 3% BSA. Copper-catalyzed azide–alkyne cycloaddition (CuAAC) was carried out for 30 min at room temperature, protected from light, using the Click-iT^™^ Protein Reaction Buffer Kit (Invitrogen, C10276) supplemented with 5 μM tetramethylrhodamine (TAMRA)-azide. Fluorescence intensity was quantified using a FACS Aria II flow cytometer (BD Biosciences). Samples were passed through a 70 μm nozzle, and cell populations were identified based on forward scatter (FSC) and side scatter (SSC) parameters. TAMRA fluorescence was detected using a 585/40 nm bandpass filter. Data were analyzed using FlowJo v10 software (BD Biosciences).

### Detection of Nt-formylated native proteins

Nα-terminally formylated proteins from *Borrelia burgdorferi* after Forazemin treatment was determined by immunoblotting with slight modifications^47^. *Borrelia burgdorferi* BbP1286 was cultured in BSK-II medium in a microaerophilic chamber (35 °C, 3% O_2_, 5% CO_2_) to late logarithmic phase. Cultures were then incubated overnight with forazemin (2xMIC; 1 μg ml^−1^); untreated cultures served as controls. Samples were centrifuged and resuspended in PBS buffer before heat-denaturation at 98 C. Protein concentrations were quantified using bicinchoninic acid assay (Thermo Fisher Scientific ) before resuspension in 1x Laemmli buffer. 20 μg of proteins were separated on 4–15 % SDS-PAGE and transferred on nitrocellulose membrane with Trans-Blot Turbo Transfer System (Bio-Rad). Mouse monoclonal Pan-Formylmethionine Antibody (ImmuneChem) were used at a 1/2000 dilution and horse radish peroxidase (HRP)-conjugated anti-mouse antibody, and signals were detected by chemiluminescence using the Clarity Western ECL kit (Bio-Rad).

### Microscopy

*Borrelia burgdorferi* BbP1286 was cultured in BSK-II medium in a microaerophilic chamber (35 °C, 3% O_2_, 5% CO_2_) to late logarithmic phase. Cultures were then incubated overnight with forazemin (10xMIC; 5 μg ml^−1^); untreated cultures served as controls. The samples were split for epifluorescence microscopy and scanning electron microscopy. Treated and control cells were allowed to adhere to poly-L-lysine–coated coverslips for 15 min and fixed in 2.5% glutaraldehyde for 1 h at room temperature. Samples were washed three times in 0.1 M sodium cacodylate buffer (pH 7.2), post-fixed in 0.1% osmium tetroxide in 0.1 M sodium cacodylate (pH 7.2) for 30 min and washed twice again in cacodylate buffer. Dehydration was carried out in a graded ethanol series (30%, 50%, 70%, 85%, 95%, 100%; 5 min each). Specimens were dried by critical point drying with liquid CO_2_ (Tousimis Samdri-PVT-3D), mounted on aluminum stubs, sputter-coated with platinum (208HRD sputter coater), and examined using a Hitachi S-4800 field-emission scanning electron microscope.

### In vitro Blood Brain Barrier assay

Human brain microvascular endothelial cells (hCMEC/D3) and primary human astrocytes were used to establish a Transwell blood–brain barrier (BBB) model^48^. Transwell inserts (polycarbonate, 0.4 μm pore size, 12-well format) were coated with collagen type I (50–100 μg ml^−1^ in PBS, 1 h at 37°C), washed with PBS, and equilibrated with endothelial growth medium. hCMEC/D3 cells were seeded on the upper (apical) surface at 10^5^ cells cm^−2^ and cultured to confluence (3–5 d, 37 °C, 5% CO_2_), with medium changed every other day.

Primary human astrocytes were seeded on the underside of the inserts or in the lower (basolateral) compartment at 5×10^4^-1×10^5^ cells per well in astrocyte medium 24 h before co-culture. Once a stable monolayer formed, media were replaced with serum-reduced BBB co-culture medium, and transendothelial electrical resistance (TEER) was monitored until plateau (typically >20–30 Ω·cm^2^ for hCMEC/D3). Forazemin and other antibiotics were added to the apical chamber at the indicated concentrations; samples were collected from apical and basolateral compartments at time points (15 to 120 min) to determine drug permeability by LC-MS/MS readout. TEER was measured before and after the assay to confirm barrier integrity, and apparent permeability coefficients (Papp) were calculated from concentration/time.

### Murine Lyme Borreliosis infection model

All animal experiments were approved by the Northeastern University Institutional Animal Care and Use Committee (IACUC, protocol number 1606–19). Five-week-old male wild-type C3H/HeNCrl mice (strain code 025; Charles River Laboratories) were infected subcutaneously in the flank with 10^5^ mid-log-phase *B. burgdorferi* N40 (clone D10E9) cells or intradermally in the ear pinnae with bioluminescent *B. burgdorferi* ML23 pBBE22luc (a lp25-deficient B31 derivative carrying codon-optimized luciferase under the *flaB* promoter; n = 5 mice per treatment group)^17,27^. Forazemin was formulated in 10% DMSO, 40% PEG300, 5% Tween-80, and 45% water (v/v/v/v).

Infection was allowed to establish for three weeks, confirmed by plating ear-punch biopsies (2-mm diameter) onto semi-solid BSK-II medium supplemented with 1.5% gelatin; growth was observed in all animals within 14 d. For bioluminescence imaging, mice received intraperitoneal injection of 5 mg VivoGlo d-luciferin (Promega) dissolved in 100 μl sterile PBS, followed 10 min later by whole-body imaging (2–5 min exposure) on an IVIS Spectrum in vivo imaging system (PerkinElmer) to quantify luminescence. Treatment began immediately after infection confirmation: mice received twice-daily oral gavage for 5 consecutive days with vehicle; untreated controls), doxycycline (100 mg kg^−1^ body weight; positive treatment control), or forazemin (10, 25 or 50 mg kg^−1^). Infection clearance was monitored by ear-punch biopsies collected every 12 h after treatment initiation (plated in semi-solid gelatin/BSK-II medium) and by whole-ear cultures at necropsy on day 21 after infection. Bacterial outgrowth was scored after 14–21 d of incubation (34 °C, microaerophilic). For overall borrelia burden, we followed microscopy enumeration described below.

### Estimation of *Borrelia* burden in mouse tissues by Dark-field microscopy

Immediately following euthanasia after treatment period, the ear pinna, bladder and/or a 6 mm punch biopsy of dorsal back skin was collected from each animal using sterile scissors and forceps, which were flamed and cooled between animals to prevent cross-contamination. Each tissue was briefly rinsed in sterile phosphate-buffered saline (PBS, pH 7.4) to remove surface blood and debris, blotted dry on sterile gauze, and weighed to the nearest 0.1 mg on an analytical balance. Tissues were then transferred to sterile 1.5 ml microcentrifuge tubes containing a fixed volume of BSK-II medium (500 μl for ear pinna; 1,000 μl for back skin biopsies and bladder) and homogenized using a bead beater (TissueLyser II, Qiagen) at 25 Hz for two cycles of 1 minute each, with a 1-minute rest on ice between cycles in the presence of a single 3 mm stainless steel bead; homogenates were maintained on ice and not centrifuged to prevent spirochete sedimentation. For enumeration, 10 μl of undiluted homogenate was loaded into a Petroff-Hausser bacterial counting chamber (depth: 0.02 mm; Hausser Scientific) by capillary action and allowed to settle for 2–3 minutes at room temperature. Spirochetes were visualized on a dark-field microscope using a 40× objective and counted across a minimum of 10 large grid squares in at least 3 independent fields of view; only motile, morphologically intact corkscrew-shaped-organisms were counted, and all samples were enumerated by two independent blinded observers. Spirochete concentration was calculated as: spirochetes ml^−1^ = average count per small square × 2 × 10^7^, and final burden was expressed as spirochetes per milligram of tissue by multiplying by the homogenization volume and dividing by tissue weight.

### Murine Neuroborreliosis model

All biohazard and animal experiments were carried out in accordance with approved protocols from the University of North Dakota Institutional Biosafety Committee (protocol number IBC2510–0054) and the Animal Care and Use Committee (IACUC, protocol number 2501–0001; Animal Welfare assurance number A3917–01), respectively. Low-passage *B*. *burgdorferi* strain B31-A3 cultured to mid-log phase in BSK-II medium at 37°C, 5% CO_2_, and quantified by dark field microscopy using a Petroff-Hausser chamber. C3HeB/FeJ (C3H; stock # 000658) were purchased from The Jackson Laboratory. 6–8-week-old male and female mice were used. For infections by needle inoculation, animals were placed under anesthesia using isoflurane inhalation, followed by subdermal inoculation with 100 μl of BSK-II medium containing 10^6^ spirochaetes. Infections were confirmed in mice by collecting ~80 μl blood from the saphenous vein on day 7 after infection and cultured in BSK-II. Ear tissue was also isolated and cultured at times of sacrifice of all animals. Dark-field microscopy was used to confirm the presence of viable spirochaetes for each cultured blood/tissue sample. Forazemin and doxycycline (Research Products International) at 100 mg kg^−1^ day^−1^ were started on day 8, twice a day (every 12 h) for 5 d. Drugs were administered via oral gavage (0.11 ml per dose) to equal numbers of male and female animals. Mice were sacrificed after 5 days of drug treatment. Skullcaps with attached dura were isolated by craniotomy and dura mater removed from the skullcaps as previously described and cultured in BSK II media. Brains were sliced into two halves, with one half cultured in BSK II medium^28^.

### Murine prophylaxis model

All animal procedures in this study were approved by the Tufts University-Tufts Medical Center Institutional Animal Care and Use Committee (IACUC, protocol number B2024–50).

#### Generation of *B. burgdorferi* infected *I. scapularis* nymphs.

Larval *Ixodes scapularis* ticks were obtained from Oklahoma State University and stored in humid airtight containers at 4°C. *B. burgdorferi* (strain B31) infected mice were infested with approximately 100 larval *I. scapularis* ticks by placing both mice and the ticks in a restraining chamber for 1 h before returning to a cage suspended in a water moat. Water moats were monitored for 10 d to collect fed larval ticks. Fed ticks were placed in microcentrifuge tubes and stored in an airtight glass desiccator containing saturated potassium sulfate at room temperature. Larval ticks were allowed to molt and were stored in humidified chambers at 4°C. Infection rate was determined by culturing five molted nymphs per batch in BSK-II medium at 32°C. Male C3H/HeNCrl mice (strain code 025; Charles River Laboratories) aged seven weeks were infected with *B. burgdorferi* either via nymphal tick infestation or subcutaneous needle inoculation^49^. Forazemin was formulated as mentioned previously. Doxycycline was resuspended in distilled water and prepared on the day of dosing. To test the efficacy of post-exposure prophylaxis, mice were infested with five infected nymphs using tick-restraining capsules positioned between the shoulder blades of the mouse as previously described^50^ . Restraints were removed after four days, and fed ticks were collected. Mice were singly housed in water-moated cages during infestation and returned to group housing after all ticks had been collected. To determine the point in time after tick bite that delivery of antibiotic was effective, a single dose of forazemin or doxycycline (100 mg kg^−1^ per dose) was administered by oral gavage at either 0, 48, or 96 h following tick removal. The efficacy of a two-dose regimen was also assessed, whereby forazemin or doxycycline (100 mg kg^−1^ per dose) was administered by two times by oral gavage in span of 24 h following tick removal. Mice were euthanized 21 d after infestation. Back, bladder, and ear tissues were cultured in BSK-II medium supplemented with amphotericin B (5 μg ml^−1^), phosphomycin (100 μg ml^−1^) and rifampicin (50 μg ml^−1^), incubated at 32°C, and monitored for 3 weeks via dark-field microscopy.

### Impact of forazemin on the murine fecal microbiome

C3H/HeNCrl mice (strain code 025; Charles River Laboratories) were infected subcutaneously with 10^5^ *B. burgdorferi* N40 cells and infection was allowed to establish for 3 weeks^17^. Mice then received vehicle (10% DMSO, 40% PEG300, 5% Tween 80, 45% water, v/v/v/v), doxycycline (100 mg kg^−1^ by oral gavage), 10, 25 and 100 mg kg^−1^ forazemin or remained untreated (n = 5 mice per group per experiments), dosed twice daily for 5 d. Fecal pellets were collected before and after treatment initiation, 13 days after start of treatment for recovery snap-frozen in PBS, and stored at −80 °C. DNA from 100 mg of mouse stool was isolated using the ZymoBIOMICS^™^ DNA Miniprep Kit following standard protocol. Quantification and QC for samples were performed via NanoDrop. Samples were sent to SeqCoast for 16S amplicon sequencing. DNA samples were prepared for 16S V3/V4 amplicon sequencing using the Zymo Quick-16S Plus NGS Library Prep Kit. Sequencing was performed on the Illumina NextSeq2000 platform using a 600-cycle XLEAP-SBS flow cell kit to produce 2×300 bp paired reads. 30–40% PhiX control (unindexed) was spiked into the library pool to support optimal base calling of low diversity libraries on patterned flow cells. Read demultiplexing, adapter trimming, and run analytics were performed using DRAGEN v4.2.7, an on-board analysis software on the NextSeq2000. Analysis was performed using QIIME 2 (2024.10 distribution). Primer sequences were trimmed using Cutadapt with 341F(CCTACGGGDGGCWGCAG/ CCTAYGGGGYGCWGCAG) and 806R (GACTACNVGGGTMTCTAATCC) primers. Sequences were denoised using DADA2 to generate amplicon sequence variants (ASVs), with removal of chimeric sequences and low-quality reads. Taxonomic classification was performed using a naïve Bayes classifier trained on the Greengenes2 (2022.10) database extracted to the V3-V4 region using the 341F and 806R primers. Alpha diversity was assessed using the Shannon Index, and Gini-Simpson index.

### Short-chain fatty acids analysis

Short-chain fatty acids (SCFAs) in murine fecal samples (n = 5) were quantified using a commercial ELISA kit (Lifeome BioLabs) according to the manufacturer’s protocol. Briefly, frozen fecal pellets (25–30 mg) were weighed and homogenized in ice-cold PBS or kit extraction buffer (1:9, w/v) using a bead mill, followed by centrifugation (10000×g, 4°C) to clarify the supernatant. Supernatants were diluted as required in sample diluent and loaded (50 per well) onto pre-coated ELISA plates alongside SCFA standards. After incubation with capture and HRP-conjugated detection antibodies and intervening wash steps, TMB substrate was added, the reaction was stopped with acid, and absorbance at 450 nm was measured on a microplate reader. SCFA concentrations were calculated from a standard curve and normalized to fecal wet weight (mg g^−1^)

### Quantification and statistical analysis

GraphPad Prism was used for statistical analysis. All statistical details for each experiment are described in figure legends. A *P* value ≤ 0.05 was considered statistically significant.

## Supplementary Material

This is a list of supplementary files associated with this preprint. Click to download.

• supplementaryinformation.docx

• ExtendedData.docx

## Figures and Tables

**Table 1.** Forazemin and clinically relevant antibiotics acting against spirochaetes, human pathogens and commensals.

	MIC μg ml^−1^)		
Organism and genotype	Forazemin	Doxycycline	Ceftriaxone
**Spirochaetes**			
*Borrelia burgdorferi* B31	0.5	0.25	0.01
*Borrelia burgdorferi* 297	1	0.25	0.01
*Borrelia burgdorferi* N40	0.5	0.25	0.01
*Borrelia bavariensis*	0.5	0.125	0.01
*Borrelia garinii*	0.5	0.125	0.25
*Borrelia afzelii* ATCC 51992	0.5	0.25	0.01
*Borrelia spielmanii* DSM 16813	1	0.125	0.125
*Borrelia hermsii* HS1	0.125	0.5	0.25
*Borrelia hermsii* DAH	0.125	0.25	0.25
*Borrelia miyamotoi* ATCC BAA-3151	0.5	0.25	0.125
*Brachyspira hyodysenteriae* B-78	0.5	1	0.01
*Leptospira biflexa* ATCC 23582	0.25	2	2
*Treponema pallidum*	0.125	0.125	0.0007
**Opportunistic pathogens**			
*Staphylococcus aureus* HG003	8	0.125	4
*Enterococcus faecalis* ATCC 29212	1	0.5	64
*Escherichia coli* ATCC 25922	128	2	0.125
*Pseudomonas aeruginosa* PAO1	128	32	64
*Moraxella catarrhalis* ATCC 25238	0.125	0.5	0.25
**Gut commensal bacteria**			
*Bifidobacterium longum*	8	0.25	4
*Ligilactobacillus salivarius*	>64	1	16
*Lactobacillus reuteri*	>64	4	32
*Bacteroides ovatus*	1	0.01	0.02
*Parabacteroides merdae*	2	0.06	0.25
*Akkermansia muciniphila*	16	0.25	0.25
*Blautia producta*	32	2	0.5
*Roseburia hominis*	32	8	2

**Table 2.** Efficacy of forazemin and doxycycline against murine neuroborreliosis.

Treatment group (100 mg kg^−1^ day^−1^)	Number of mice with positive culture/number of mice tested	
	Brain	Dura mater
NT	0/4	4/4
Doxycycline	0/6	0/6
Forazemin	0/6	0/6

**Table 3.** The efficacy of forazemin and doxycycline as tick bite prophylaxis agents.

Treatment group (100 mg kg^−1^)	Number of mice with positive culture/number of mice tested		
	Ear	Back	Bladder
Single dose prophylaxis			
Doxycycline, post tick bite			
Immediately	3/3	3/3	3/3
48 h	4/4	4/4	4/4
96 h	4/4	4/4	4/4
Forazemin, post tick bite			
Immediately	3/4	3/4	3/4
48 h	2/4	2/4	2/4
96 h	2/4	2/4	2/4
Two dose prophylaxis			
First dose immediately; second dose 24 h post-bite			
Doxycycline	8/8	9/9	9/9
Forazemin	0/9	0/9	0/9
Control	9/9	9/9	9/9

## Data Availability

Data supporting the findings of this study are available within the paper with source data and extended data, supplementary information. 16S rRNA amplicon sequencing data generated in this study will be deposited in the NCBI Sequence Read Archive (SRA) under BioProject accession number PRJNA1445310 and will be publicly available upon publication. Any other data or datasets from the current study are available upon reasonable request to the corresponding author. The structural model of *Borrelia burgdorferi* peptide deformylase (BbPDF) in complex with forazemin generated using the Boltz-2 algorithm is available from the corresponding author upon request. The crystal structure of *Leptospira interrogans* peptide deformylase used for comparative docking analysis is publicly available in the Protein Data Bank under accession code 1SZZ. Non-commercial *Borrelia* and all other bacterial strains, drug forazemin used in this study are available from the corresponding author upon request, subject to a material transfer agreement where applicable.
